# The Multi-Omic Approach to Newborn Screening: Opportunities and Challenges

**DOI:** 10.3390/ijns10030042

**Published:** 2024-06-21

**Authors:** Alex J. Ashenden, Ayesha Chowdhury, Lucy T. Anastasi, Khoa Lam, Tomas Rozek, Enzo Ranieri, Carol Wai-Kwan Siu, Jovanka King, Emilie Mas, Karin S. Kassahn

**Affiliations:** 1 Department of Biochemical Genetics, SA Pathology, Women’s and Children’s Hospital, Adelaide, SA 5006, Australiatomas.rozek@sa.gov.au (T.R.);; 2 Department of Molecular Pathology, SA Pathology, Adelaide, SA 5000, Australia; ayesha.chowdhury@sa.gov.au (A.C.); lucy.anastasi@sa.gov.au (L.T.A.); 3 Adelaide Medical School, Faculty of Health and Medical Sciences, The University of Adelaide, Adelaide, SA 5000, Australia; 4 Immunology Directorate, SA Pathology, Adelaide, SA 5000, Australia; 5 Department of Allergy and Clinical Immunology, Women’s and Children’s Hospital, Adelaide, SA 5006, Australia; 6 Discipline of Paediatrics, Women’s and Children’s Hospital, The University of Adelaide, Adelaide, SA 5006, Australia

**Keywords:** newborn screening, multi-omics, mass spectrometry, genomic screening, dried bloodspot, public acceptability, metabolomics, whole-genome sequencing

## Abstract

Newborn screening programs have seen significant evolution since their initial implementation more than 60 years ago, with the primary goal of detecting treatable conditions within the earliest possible timeframe to ensure the optimal treatment and outcomes for the newborn. New technologies have driven the expansion of screening programs to cover additional conditions. In the current era, the breadth of screened conditions could be further expanded by integrating omic technologies such as untargeted metabolomics and genomics. Genomic screening could offer opportunities for lifelong care beyond the newborn period. For genomic newborn screening to be effective and ready for routine adoption, it must overcome barriers such as implementation cost, public acceptability, and scalability. Metabolomics approaches, on the other hand, can offer insight into disease phenotypes and could be used to identify known and novel biomarkers of disease. Given recent advances in metabolomic technologies, alongside advances in genomics including whole-genome sequencing, the combination of complementary multi-omic approaches may provide an exciting opportunity to leverage the best of both approaches and overcome their respective limitations. These techniques are described, along with the current outlook on multi-omic-based NBS research.

## 1. Introduction

Newborn bloodspot screening (NBS) has been a very successful public health program since its inception in the early 1960s when screening for phenylketonuria was first introduced. Today, NBS is offered in many countries, but there are significant differences between countries and even jurisdictions in the conditions screened for [1,2,3]. In Australia, NBS programs commonly screen for congenital hypothyroidism, galactosaemia, cystic fibrosis (CF), amino acid disorders, fatty acid oxidation disorders, organic acid disorders, congenital adrenal hyperplasia, spinal muscular atrophy (SMA), and severe combined immunodeficiency (SCID). The latter two conditions were only recently introduced into Australian screening programs with efforts to further expand the number and types of conditions screened [4].

The selection of conditions for NBS is guided by rigorous processes with consideration of clinical, ethical, economic, and technical factors, and is still broadly guided by principles originally proposed by Wilson and Jungner in 1968 [5]. Advances in technology, especially in the areas of metabolomics and genomics, mean that it may soon be technically feasible to screen for many more conditions. How to most effectively and appropriately apply these technologies for the expansion of NBS programs is hence a very active area of research. The International Consortium for Newborn Sequencing (ICoNS) aims to connect research groups and provide a collaborative platform to develop best practices among genomic NBS (gNBS) programs.

In 2021, the Australian Government through the Medical Research Future Fund announced a targeted call for research to explore models of gNBS. Five research programs were funded to explore research questions around feasibility, scalability, ethics, policy, cost-effectiveness, and public acceptability. These research projects collaborate in a national consortium, the Genomic Screening Consortium for Australian Newborns (GenSCAN) [6]. Our research program, NewbornsInSA [7], uses a multi-omics approach to explore the feasibility and opportunities offered by combining metabolomics and genomics into a single NBS strategy.

In this review, we describe current advances in metabolomics and genomics and discuss opportunities and challenges in their application to NBS (Table 1).

## 2. Metabolomic Approaches to NBS

Metabolomics refers to the study of low-molecular-weight molecules (typically below 1500 Da) produced by cells and organs, such as lipids, carbohydrates, amino acids, and organic acids. These molecules, known as metabolites, can be important phenotypic biomarkers of disease [8,9]. Through variations in sample preparation and analytical methods, metabolomics can assess a suite of metabolites with highly variable physical properties ranging from large and non-polar to smaller and polar compounds [10]. Post-extraction filtration is also utilized to separate extracted analytes of interest [54].

The introduction of tandem mass spectrometry (MS/MS) has been a ground-breaking change in how screening was conducted to identify inborn errors of metabolism (IEMs) [55]. A key technique for NBS since the 1990s, MS/MS provided a method to detect multiple metabolites of varying chemical properties in a single assay and reduced the incremental cost of adding new tests, compared to the traditional model of ‘one test–one disorder’ [56,57]. A multiplexed assay based on this technology, which measured amino acids and acylcarnitines, enabled the expansion of screening to include a number of amino, organic, and fatty acid disorders using a single blood spot, with a reasonable cost and rapid turn-around time [58].

This possibility of expanded NBS offered by MS/MS approaches sparked much discussion, and even controversy, regarding which additional conditions should be screened for [59,60]. Questions around benefit versus harm, benefits outside immediate treatment, minimizing false-positive results, result communication and follow-up were raised. In an effort to standardize NBS programs across the US, the Recommended Uniform Screening Panel was established [61]. Australia has also committed to standardizing screened conditions across states and territories [4].

The combined application of liquid chromatography (LC) and MS/MS enabled further expansion of the number of screened conditions. LC-MS/MS has been applied for both first- and second-tier testing, offering significant benefits compared with MS/MS alone in detecting a broader range of conditions, particularly lysosomal storage disorders [62]. Whilst more targeted tests continue to be added to the repertoire of LC-MS/MS capabilities, the next frontier may be in exploring untargeted metabolomic approaches for use in NBS.

Untargeted ‘shotgun’ metabolomics is predominantly performed by directly introducing the sample mixture into high-resolution mass spectrometers such as quadrupole time-of-flight mass spectrometers (QTOF-MS) or tandem orbitrap instruments through flow injection. Other approaches include partial targeting of hydrophobic and hydrophilic species by using different types of separation techniques. In a single experiment, QTOF-MS instruments can provide a high-resolution survey of metabolites through the acquisition of primary MS data over a wide mass/charge range. Information-dependent acquisition scans are also utilized to fragment molecules, providing additional structural information [63]. Using untargeted metabolomics, thousands of non-specific features can be detected in a sample. Bioinformatic analyses are performed to identify outlying metabolite concentrations in specific patient samples when compared with a larger control cohort [24,64,65].

### 2.1. Opportunities for Metabolomic NBS

Due to the ease of sample collection, the small volume of blood required, and the ability for samples to be stored at ambient temperature for extended periods of time, dried blood spots (DBSs) are the standard sample used for NBS programs across the world [13,66,67]. In addition, DBSs have been shown to contain some metabolites not present in plasma samples. Ottosson et al., reported that after 10 years of storage, more than 70% of the metabolome remained intact in DBS samples, suggesting that these are well suited for retrospective epidemiological studies using untargeted metabolomic profiling [17].

Given that current NBS programs already use MS techniques, further expansion using MS/MS approaches could be readily achieved by building on existing techniques and infrastructure. MS approaches are certainly suitable for processing hundreds or thousands of samples at relatively low cost and time investment, especially when the markers of interest are known and have been well characterized [11]. For targeted analysis where compounds of interest have already been selected, triple quadrupole mass spectrometers are valued for their ability to use multiple reaction monitoring to profile for specific analytes [68].

It may be argued that compared to the genome, the metabolome provides a closer correlation to the phenotype and may give a more holistic indication of disease states as it reflects the integration of genetic variation, gene expression, protein interactions, and upstream regulatory processes [21,22]. Importantly, however, factors such as the timing of sample collection, feeding regimens, and gestational age need to be carefully controlled for, as these can alter metabolic profiles, even in the absence of an underlying genetic condition. In contrast, genetic variants are typically present at birth and not altered by sampling factors, yet their significance for disease risk can at times be difficult to ascertain. Genetic modifiers, incomplete penetrance, and variable expressivity can result in the same pathogenic DNA variants causing different disease severity or patterns of onset, even within a single family. In the context of NBS where there is no clinical phenotype, having some phenotypic read-out, such as a metabolomic profile, is thus attractive.

Untargeted metabolomics may offer the opportunity to screen for a wide range of IEMs, and likely other disorders, in a single test. A pilot study by Miller et al., used an untargeted metabolomic workflow to retrospectively screen for 21 different IEMs in a cohort of patients with known IEMs and successfully identified 20 of the 21 conditions [23]. Whilst these were not as sensitive nor specific as quantitative assays developed specifically for a given condition, they postulated that an untargeted approach such as this could be used as an initial screen for a wide range of IEMs and for novel biomarker discovery. This approach is dependent on prior knowledge of relevant metabolic pathways and the disorder causing significant enough analyte perturbations that they are detectable by current instruments.

Similarly, proof-of-principle studies have demonstrated that untargeted metabolomic studies using high-sensitivity QTOF-MS can identify known biomarkers related to specific conditions [18,19,20]. The next step in the application of untargeted metabolomics is the determination of previously unknown biomarkers in patients without knowledge of an existing diagnosis; that is, using untargeted metabolomics as a routine screening test with the possibility of adoption in NBS programs. At present, however, few NBS laboratories possess QTOF instruments, and with the complexity in sample processing and data interpretation, these types of investigations are at present firmly in the realm of research. There could be potential opportunities in combining untargeted discovery with targeted approaches. The adoption of such approaches in NBS will likely require laboratories to invest in new equipment and recruit staff with the relevant expertise, including skills in advanced statistics and modern mass spectrometry technologies.

### 2.2. Challenges for Metabolomic NBS

As thousands of features are measured in each sample, untargeted metabolomics results in extremely large datasets that are complex to interpret [69,70]. To use these data in a routine manner, their analysis must be streamlined. One approach used by Haijes et al., was to develop a method for the automated interpretation of metabolomic data using a knowledge-based algorithm. This algorithm used pre-defined weighting scores for metabolites across a range of IEMs and the sample’s metabolic profile to produce a ranked list of differential diagnoses. Differential diagnoses included the correct diagnosis for 70–80% of samples within various training sets, with the correct IEM ranking in the top three most likely conditions 57% of the time. When used in combination with the judgment of laboratory specialists, such knowledge-based algorithms could be used to determine the recommended second-tier IEM testing [37].

The application of network- and graph-based approaches to data interpretation in metabolomics is another active area of research [38], as are approaches to apply machine learning to reduce false-positive results [69]. The establishment of reference ranges for a suite of metabolites in healthy newborns [71], and the development of new software tools, including collaborative interpretation tools such as Collaborative Laboratory Integrated Reports and the Human Metabolome Database, are also helpful for data interpretation [9,72].

The interpretation of metabolomic profiles typically requires comparison against known references, and metabolomic profiling alone is not currently a diagnostic test. Additional testing is required to establish a clinical diagnosis. There are also instances in which a metabolite identified during screening may indicate the possibility of not just one, but several conditions, some of which may be non-target conditions. Some non-target conditions are of limited value for reporting in the newborn screening context, such as 3-Methylcrotonyl-CoA carboxylase deficiency, a benign biochemical phenotype with most individuals being asymptomatic, detected through the elevation of 3-hydroxyisovalerylcarnitine (C5OH) [73]. On the other hand, some non-target conditions may have health implications for the baby; for example, propionyl carnitine (C3) is the marker used to screen for methylmalonic acidemia and propionic acidemia but it also detects acquired Vitamin B12 deficiency as a non-target condition [74]. With the introduction of broader, untargeted metabolomic investigations, the risk of identifying non-target conditions may be increased. The use of specific markers and refinement of the screening algorithms may help address this challenge and avoid the ethical dilemmas associated with the detection of non-target conditions [75].

Traditionally, MS-based approaches have been applied to many IEMs. These metabolic conditions may be expected to result in measurable metabolite changes, even shortly after birth. For other conditions, such as neurological conditions and immunodeficiencies, for example, it seems less clear whether metabolites could indicate disease. However, the success of metabolomics in identifying biomarkers in non-IEM conditions, including neurogenerative diseases [76,77,78], uterine diseases [79], and bleeding disorders [80], is encouraging and may suggest that metabolomics could have broad utility, even in non-IEM conditions. For NBS, the next frontier will be in determining which condition groups have metabolite changes that can be detected in pre-symptomatic disease shortly after birth.

In the current paradigm, the addition of new biomarkers for additional diseases to NBS programs is not trivial. The expansion of NBS programs necessitates rigorous validation for each additional condition, with pre-expansion training and validation sets required to undergo preliminary testing, and the development of clear reference ranges for each biomarker. For conditions where biomarkers cannot be robustly detected or cut-off ranges for abnormal results are ill defined, metabolomic NBS may not be appropriate [39,40]. Multiplex screening by MS does significantly decrease the complexity of adding new conditions to NBS programs, but the validation requirements and the associated costs for adding new conditions remain [41].

Untargeted metabolomic testing is clearly a powerful driver for novel IEM discovery. However, a major focus until now has been on single-subject analyses, often with low cohort numbers of both known and unknown cases. Using untargeted testing to its full potential would require a sufficiently large validation cohort as a normalized population for prospective samples to be measured against [42,70].

In summary, both targeted and untargeted MS-based testing of DBSs offer many exciting opportunities for their application to NBS, but there are challenges and limitations to their use as a singular detection method in NBS. To date, second-tier testing has mostly consisted of biochemical assays; however, advances in next-generation sequencing (NGS) and genomics have made this an attractive alternative due to the improved throughput and decreased cost of implementation [81].

## 3. Genomic Approaches for NBS

At present, only a few standard NBS assays measure DNA as an analyte. These include molecular screening for SMA and SCID, which have been introduced into Australia’s NBS programs over the last 5 years [82]. Genetic testing is performed as a second-tier test for most other conditions currently included in NBS programs and is used to confirm a diagnosis and to inform the genetic risk of other family members [83]. DNA analysis is performed using a range of molecular techniques, including Sanger sequencing, quantitative real-time PCR, multiplex ligation-dependent probe amplification, and more recently NGS. NGS has now become an integral part of the screening algorithm for cystic fibrosis in many countries [83]. The implementation of NGS gene panel testing, whole-exome sequencing (WES) and whole-genome sequencing (WGS) in clinical diagnostics has sparked interest in exploring the use of these genomic approaches for NBS more broadly. Although sequencing costs remain high, WGS has been shown to increase the diagnostic yield and improve clinical actionability in patients compared to WES [12]. For this reason, WGS is being considered in many gNBS research studies as a way to maximize the lifetime utility of gNBS data.

Targeted gene panels typically use PCR amplification, inversion probes, or exon-capture techniques to selectively target and sequence genes of interest. In WES, probes are designed to capture every protein-coding exon in the human reference genome. In the case of WGS, both coding and noncoding regions are sequenced and analyzed without prior amplification or exon-capture steps. As the target size increases so does the sequencing cost, with WGS currently costing approximately AUD 1000 per human genome [84]. A comprehensive bioinformatics pipeline for the analysis of WGS will detect >5 million variants across the entire genome, including small variants and insertion/deletions, copy number variants (CNVs), structural variants, canonical splice and deep intronic variants, short tandem repeats, and mitochondrial variants. Importantly, in a gNBS context, variants can be bioinformatically filtered to selectively include only variants in genes from a pre-curated virtual NBS gene panel. Furthermore, variant prioritization and interpretation can be automated to only return pathogenic and clinically actionable variants in NBS genes of interest [85].

### 3.1. Opportunities for gNBS

Genomic NBS provides an opportunity to screen for a broader range of conditions than is currently possible using standard biochemical tests whilst using a singular methodology [31]. Thus, gNBS provides a ‘one-stop-shop’ to screen any genetic condition of interest, although there are some gene regions and variant types that remain difficult to analyze. Out of 300,000 babies screened every year in Australia in current NBS programs, an estimated 1 in 1000 are diagnosed with one (or more) screened conditions [86]. Including genomic approaches in NBS programs could significantly bolster the capacity to detect a wider range of conditions. At present, over 600 conditions are being discussed for inclusion in gNBS programs [26,27,28,29]. As new conditions are proposed for inclusion, the virtual panel for analysis can be updated immediately with minimal additional cost and validation.

Genomic screening provides an up-front molecular diagnosis. Confirmatory testing using a different testing method is recommended to establish a clinical diagnosis. An early molecular diagnosis can provide many benefits not just for improved condition management and surveillance for the newborn themselves, but also for the management of at-risk relatives and for prenatal testing of future pregnancies [26,45]. Using a Delphi approach, Kingsmore et al., retrospectively modeled the extent to which symptoms could have been avoided completely, mostly or partially, if genomic screening had been offered at birth [26]. A timely molecular diagnosis facilitates early access to precision medicine including gene therapy, small-molecule and nucleic acid drugs, enzyme replacement therapy, stem cell therapies, and multidisciplinary supportive care [30,31,32]. Many of these interventions require a molecular diagnosis to match therapy to the specific variants identified in the newborn. A timely molecular diagnosis also reduces the need for expensive and invasive tests later in the newborn’s life. Finally, samples for gNBS such as cord blood could be collected immediately after birth, thus avoiding any delay in screening, although a new clinical pathway for sample collection would need to be established.

Stored genomic data can act as a lifetime health resource for families as the data would be readily available if required for re-interrogation later in life [31]. Re-analysis may be performed to investigate new symptoms, answer different clinical questions, or for other screening applications, including pharmacogenetic applications, cancer predisposition, and reproductive carrier screening [33]. While it seems ideal from various screening perspectives to re-analyze these stored data against emerging knowledge, regular reviews would need to be incorporated into healthcare pathways and resourced accordingly. In contrast, data review with respect to a specific diagnostic or clinical question could be more readily integrated into existing diagnostic testing pathways. As stored genomic data are available immediately, there is no need for new sample collection or sequencing, which can expedite clinical review and fast-track clinical care. As stored genomic data can be analyzed multiple times for different clinical questions, it could potentially provide a very cost-effective approach for lifelong care [87].

Genomic data stored under appropriate consent and data sharing agreements can be utilized in de-identified research to improve our understanding of gene variants and their contribution to disease as well as aiding in the development of novel therapies [31,34]. Furthermore, stored genomic data from gNBS could help expand the representation of ethnic groups in global population databases and improve the accuracy of variant interpretation [33]. Presently, many genomic population databases are disproportionately skewed towards European populations. As NBS is provided at a population scale in many countries, it could help address the inequality reflected in population databases by capturing major and minor population-specific alleles [33]. With an appropriate consent process, these data could be further used in research to capture pharmacogenetic diversity driven by pharmacogenomic variants. This could enable effective ‘genotype-guided’ dosing tailored for diverse populations [35,36].

### 3.2. Challenges for gNBS

While gNBS studies could theoretically report on all the 6000+ known disease-causing genes [88], current gNBS research programs typically restrict reporting to genes and conditions that are serious, early-onset (<5 years of age), have available treatments or interventions, and where the screening test has high clinical and technical validity [28,29]. Unsurprisingly, there is currently little consensus about which genes and conditions meet these general criteria, with different interpretations of the criteria and differences in the availability of treatments across jurisdictions [29]. A recently published online compendium of genetic conditions and their treatments has assisted the curation of many emerging gNBS gene lists [43].

Genomic NBS at a population scale also increases the chances of identifying variants causing untreatable or adult-onset conditions outside of the scope of NBS [44,45]. This is particularly problematic for genes that have both autosomal dominant (AD) and autosomal recessive (AR) presentations. One example is the BRCA1 gene, for which the AR condition Fanconi anemia may be reportable in a gNBS context, but the AD susceptibility to breast cancer would not be. Some have argued for the return of such variants and carrier status information [44,89], although this contradicts current best-practice guidelines that recommend genetic tests are only provided to children if they have a direct health benefit to the child themselves [90]. The child’s autonomy and the psychosocial consequences for the parents and child later in life must be carefully considered when determining the return of variants for adult-onset conditions, and screening for conditions for which no treatment exists [91]. Others have recommended the inclusion of genes associated with neurodevelopmental diseases that currently have no available curative treatment, but could facilitate the child’s access to early interventions, clinical trials, and therapies to improve their quality of life [92]. The question of what constitutes an effective treatment or intervention is likely to cause much debate in the curation of conditions for gNBS. While the original Wilson and Jungner criteria [5] have been enduring, some have proposed a revision and expansion of the original guidelines to better reflect modern system requirements [93,94].

Population-level genomic screening, including gNBS, identifies many novel variants. It should be noted that many populations are currently under-represented in genomic databases. Without supporting literature or diverse population data, and sometimes with conflicting predictive algorithms, their clinical significance is difficult to interpret [24,47,85,95]. In the absence of a clear phenotype in an apparently healthy baby with no known parental or family history, these variants remain classified as variants of uncertain significance. In a screening context, the follow-up or functional characterization of such variants is not feasible [47,48]. As a consequence, only well-supported pathogenic variants are typically reported in gNBS programs [24,26,47]. For some conditions, the presence of both early-onset, severe forms of the disease and later-onset, milder forms of disease complicates variant interpretation and reporting further. Pompe disease is one such example, with both a late-onset and a more severe infantile-onset form [46].

Genomic NBS may have low diagnostic yield for some conditions. Some genes or variants are difficult to assay using current NGS and bioinformatic methods. For example, the F8 gene has a common inversion and the STRC gene has a near-identical pseudogene, pSTRC [50], which makes these genes more difficult to analyze. In two recent studies, NC NEXUS and NBSeq, the sensitivity of WES gNBS was comparatively high for IEM (88%), similar to that modeled by Kingsmore et al., for other genetic conditions [26], compared to a mere 18% for newborns with hearing loss conditions [49,96]. Technical limitations identifying the second variant in AR conditions, lack of variant phasing information, and difficulties interpreting clinically significant variants in a predictive setting were some of the challenges reported [49]. For other conditions, trio sequencing, whereby the newborn and parents are sequenced, is required to effectively interpret pathogenic variants. When using targeted approaches such as gene panel or exome sequencing, CNVs and intronic variants may not be detectable, leading to poor pick-up for conditions where such variants are the main drivers of disease. Lastly, the GRCh38 human reference sequence is likely not representative of diverse ethnic populations, leading to sub-optimal variant detection [97].

Providing genetic counseling at a population scale can be challenging and likely requires a different approach than that which is offered in a clinical diagnostic setting. Pre-test counseling may be offered using electronic education materials, videos, eConsent, and electronic decision support tools. However, given the broader spectrum of screened conditions, gNBS will also lead to an overall increase in newborns who screen positive. Post-test genetic counseling for families with positive screening results will likely require telehealth or in-person appointments to discuss these findings and the next steps [52]. Post-test counseling methodology must ensure parents understand that confirmatory testing is required to establish a definitive diagnosis, and that there is the possibility of the condition never developing in the child [51]. With an increased need for genetic counseling services, there is an increased likelihood that nongenetic specialists may deliver genetic counseling services with possible adverse long-term outcomes [52]. Nongenetic specialists may misinterpret screening results, offer cascade testing before arranging confirmatory tests, and provide insufficient psychosocial support to parents [31,52]. One approach to address the limited availability of genetic counselors is to provide ‘telegenetics’ whereby structured phone interviews or video-based technologies are used to communicate with patients more efficiently [52].

Genomic data generated at a large scale, with an appropriate consent process, are theoretically reusable for healthcare and research purposes [98]. In practice, however, storing and managing genomic data and consent at scale is challenging. While health data are commonly stored in electronic health records (EHRs), integrating genomic data into EHRs is currently difficult due to issues with storage capacity, data linkage, interoperability, and privacy concerns [99]. Genomic data may be stored as variant call files rather than alignment or raw data files, thus reducing the total file size; however, this restricts the scope for future analyses [100]. Privacy concerns are associated with the re-identifiability of genomic data due to the presence of rare and private variants [99]. The protection of stored genomic data against access by third parties, including government agencies outside of health, such as law enforcement agencies, needs to be carefully considered and managed. Storage of genomic data will also need to consider best practices and appropriate security measures for cloud-based and on-premise storage solutions. Some jurisdictions require storage to occur locally, rather than overseas. Privacy breaches or inadvertent disclosure of private genomic information could result in discrimination by health insurance companies, social stigma, and unwanted discovery of genetic predispositions [101]. For the research use of stored genomic data, the management of dynamic consent and consenting to multiple individual projects can become exhausting, costly, and overwhelming for both participants and researchers.

The feasibility of rapid genome sequencing has been explored in a diagnostic setting for critically ill children [102,103,104]. For application in NBS, genomic results must be returned within a few weeks to avoid missing the window of therapeutic opportunity [53]. This can be logistically challenging as some DBS samples fail library preparation, have insufficient DNA, or require additional sequencing to meet the minimum depth of coverage and QC metrics for variant calling. Turn-around time is also dependent on the frequency of sequencing and the capacity of the NGS platform [105]. Finally, confirmatory testing adds to the total analysis time. The optimization and automation of laboratory workflows as well as analysis pipelines will be critical to meet the turn-around time required for gNBS.

One of the criteria outlined by the Wilson and Jungner screening guidelines is that a ‘test should be acceptable to the population’ [5]. The public acceptability of gNBS revolves around family, health practitioners, and the general public’s perspectives [14,15]. Lynch et al., reported that parents and the general public held positive attitudes towards gNBS due to its benefits associated with providing an early diagnosis and a rich resource for advancing genomic research [14]. However, positive attitudes were largely dependent on the quality of the consent process and how well potential risks were communicated to parents [14]. Parental support for gNBS was positive, provided that education and support was offered prospectively by health professionals to guide the informed consent process. This allowed parents to better understand the screened conditions offered and the safety of their child’s genomic data [14]. In current NBS programs, parents are provided with information about screening in late pregnancy and consent for NBS is sought at the time of collecting the heel-prick sample. It is not uncommon for parents to report, in hindsight, limited recollection of the actual consent process or understanding of the screening program [106]. When considering genomic screening, models to ensure informed consent need to be carefully developed; this will avoid parents participating without understanding the implications of gNBS. Genomic NBS studies thus typically apply an ‘opt in’ consent model in which parents actively enroll for genomic screening.

Casauria et al., reported that parents also expressed favorable attitudes towards expanding the list of conditions to include common, non-communicable conditions regardless of the age of onset, such as the risks of developing cancer, cardiovascular diseases, and type 2 diabetes [14,16]. Parents suggested that early screening for these conditions could facilitate the adoption of healthier lifestyles for their child, ultimately reducing the likelihood of developing these conditions [16].

Despite general positive attitudes towards gNBS, most parents were less willing to participate in a program that included genomic sequencing compared to traditional NBS. One reason for this was the informed consent process for gNBS, which can be challenging and complex in terms of decision-making. New parents may feel overburdened with additional details and may be more likely to opt out of research studies that include gNBS if the consent process is too rigorous and extensive, potentially reducing participation rates in gNBS [14,16,107].

In a survey conducted in 2016 by White et al., Australian health practitioners felt it was too early to introduce genomic sequencing into NBS but believed that it should be incorporated within the next decade. This conservative support was mainly due to their concerns around informed consent, insurance, and disability discrimination based on genetic information, parental knowledge about gNBS limitations, the newborns’ autonomy, and genomic equity [15]. In a more recent survey conducted in 2022 by Gold et al., there was widespread support among rare disease experts for gNBS with over 87% agreeing that it should be made available to all newborns [108]. There was, however, much less agreement about which conditions should be included with only 25 genes being supported by at least 85% of experts.

Currently, there is near-universal support for NBS; therefore, any policies to implement genomic sequencing in NBS should consider parental values and address concerns raised by health practitioners to maintain high levels of NBS participation. To ensure parents still maintain their support for traditional NBS whilst not being dissuaded by the genome screening of newborns, gNBS could be offered as an optional add-on to traditional NBS. This would require explicit informed consent with an opportunity for pre- and post-test genetic counseling for parents to help understand the results as well as the utility and limitations of gNBS [107].

## 4. Multi-Omic Approaches to NBS

Genomics and metabolomics both offer exciting opportunities for application in NBS programs, but each approach also has significant limitations. While metabolomics may offer important insights into a newborn’s early disease phenotype, current techniques still require extensive validation and selection of relevant biomarkers or features for analysis. On the other hand, genomics could offer a single test to screen many conditions with limited individual validation, but in the absence of a phenotype, genetic variants are often difficult to interpret. It is thus unlikely that either approach would provide a single comprehensive solution to expand NBS in the future. The integration of genomics and metabolomics into a multi-omics program warrants further consideration. By integrating multiple, complimentary omic techniques, a multi-omics approach may provide a broader understanding than is possible with only one technique alone.

Multi-omic research into solving disease-related problems can be approached from different directions. A genome-first approach begins at the genomic level and then incorporates other omic types, whilst a phenotype-first approach may begin with a proteomic, metabolomic, or phenomic approach. The combination of multiple omic technologies could provide a more holistic understanding of factors contributing to disease pathways [109].

Multi-omic approaches have been successful in various healthcare and biomarker discovery research studies, including in the detection of risk factors for complex disease [110], cancer research [111], and infectious disease research [112,113]. In rare disease diagnostics, Coene et al., developed a method to perform high-resolution untargeted metabolomics using a single platform, which they termed ‘next-generation metabolic screening’. With the use of QTOF-MS, they detected more than 10,000 features within plasma samples from patients with 46 different IEMs. They were able to correctly diagnose 42 of the 46 IEMs using statistical analyses to determine the peaks of significant perturbation from the mean, calculated from a pool of control samples. As a secondary step, the untargeted metabolomics data went through a further round of data analysis, this time untargeted towards any known IEM perturbations. Untargeted analysis uncovered a new biomarker related to histidinemia, as well as the novel diagnosis of N-acetylneuraminic acid phosphate synthase deficiency when used in combination with exome sequencing [24,25]. Some rare disease databases are now set up to specifically capture multi-omics data, including genomic, transcriptomic, epigenomic, metabolomic, and phenotypic data, with one such example being the Genomics4RD database set up by the Care4Rare Canada Consortium [114].

A review of the omic literature by Zhang et al., found that whilst many studies have been performed on DBS using either metabolomic, genomic, epigenomic, or proteomic techniques, very few studies have used DBS for multi-omic investigations [115]. One such study was by Kerhofs et al. [116]. They performed untargeted metabolomics on DBSs from 97 patients known to have 46 different IEMs to successfully create a prioritized list of possible disease-causing genes with which variants from whole-exome sequencing could be prioritized and interpreted [116]. Similarly, Almeida et al., used an integrated multi-omics approach of first-tier gene panel sequencing with biochemical testing to improve the diagnostic rate of IEMs in blood and DBS samples [117].

**Figure 1:**
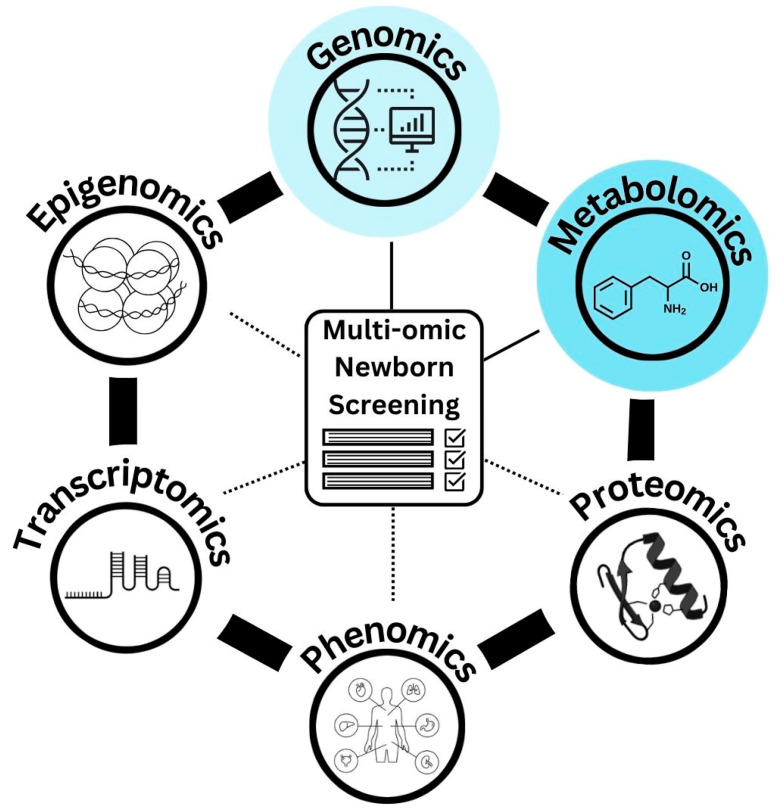
Integration of omics approaches into a multi-omics newborn screening program, with metabolomic and genomic NBS research being the focus of this review.

In an NBS setting, an integrated multi-omic approach combining both metabolomic and genomic techniques on DBS samples may overcome the challenges of using either first-tier genomic or metabolomic screening alone (Figure 1). In a cross-sectional analysis conducted between 2014 and 2019, Liu et al., compared the screening capabilities of traditional metabolic tests with metabolomic screening that integrated sequencing and clinical data. They found that metabolomic screening had a sixfold higher diagnostic rate for IEMs compared to traditional metabolic and biochemical screening and identified a wider range of IEMs. The authors cited several limitations, including the inability to screen larger metabolites which are likely significant for some disorders, and a turn-around time of 14–21 days for results which limits its viability for acute metabolic interventions. Nevertheless, the study demonstrated the potential that multi-omics approaches could have in a screening context [64]. Novel algorithmic approaches, such as machine learning tools, may be required to derive the maximum benefit from the analysis of multi-omics NBS data [118].

In the future, other omics technologies may be considered for application in NBS programs (Figure 1). Epigenomics is currently being explored in an NBS context to detect chromosome 15 imprinting disorders, including Angelman, Prader–Willi, and chromosome 15 duplication syndromes [119]. A promising area of research is also the integration of proteomics with genomics to assist in genetic variant prioritization and interpretation [120]. Similarly, transcriptomic approaches can assist in assessing the pathogenicity of rare variants [103], although—to our knowledge—these approaches have not yet been explored in an NBS context. Population-scale studies are necessary to assess the feasibility, acceptability, and sustainability of multi-omic techniques as part of NBS programs. In our research program, NewbornsInSA, we seek to address some of these questions by applying an untargeted metabolomics first-tier screen to an unselected, prospectively recruited population of up to 40,000 newborns. DBS samples with abnormal metabolomic first-tier scores will be offered second-tier WGS. Metabolomic and genomic data will be integrated to assist in the interpretation of the clinical significance of any identified genetic variants. In addition, concurrent genomic and untargeted metabolomics NBS will be offered to newborns who present with risk factors during pregnancy or soon after birth, such as abnormalities identified on antenatal scans or screening results, or high-risk clinical presentations. We hope our research program may assist in informing about the relative merit of a first-tier genomics or first-tier metabolomics approach and the strength of integrating multi-omics data for NBS. Initial data on the cost-effectiveness and acceptability of these respective approaches will be key to informing the future direction of gNBS research and ultimately the translation of gNBS into policy and practice.

Finally, the routine assessment of a newborn includes a number of clinical assessments, including a physical examination to identify critical congenital cardiac disease, congenital hip dysplasia, and abnormal hearing. While this review focused on the newborn blood spot screening programs and their possible extension using multi-omics, a truly comprehensive screening program would seamlessly integrate all these complementary screening approaches. For example, many hearing loss conditions are included in gNBS gene lists. Integrating results from newborn hearing screening may assist in the interpretation of gNBS data for these conditions. Furthermore, newborn screening sits alongside other screening programs, including pre-conception screening and prenatal screening. In Australia, a three-gene pre-conception screening for cystic fibrosis, SMA, and Fragile X, was introduced into routine clinical care in November 2023, with expanded reproductive carrier for over 750 conditions also being considered for implementation, after having been explored in a research setting [121]. Similarly, antenatal screening in Australia includes screening for chromosomal abnormalities and, in some jurisdictions, screening for hemoglobinopathies. Prenatal genomic testing following from abnormal ultrasound findings is also being offered in multiple jurisdictions [122]. These types of programs are complementary to NBS on DBS and offer different opportunities for preventative health and early clinical management.

## 5. Conclusions

Genomic and metabolomic technologies, both as individual entities and as a combined, multi-omic approach, offer exciting opportunities for expanding the scope of NBS programs into the future. Despite showing great promise for future applications in this context, both technologies have significant limitations which must be evaluated and overcome prior to implementation in routine screening programs. NBS programs have been a highly successful public health initiative, and it is essential to maintain this through a rigorous evaluation of the feasibility and acceptability of any new testing approaches. It is imperative that any changes to NBS programs do not erode the extremely high level of public acceptability they currently enjoy. Predictive testing in asymptomatic newborns poses significant ethical challenges with respect to the desire to do no harm and concerns regarding the overmedicalization of newborns, and unnecessarily alarming parents. The maximum benefit from these technologies may be derived from applying a multi-pronged approach that leverages information from multiple-omics data to more accurately predict disease risk and thus limit the risk of over-interpreting omics screening results. The overall effectiveness of a multi-omic NBS approach, and how this might align with the current standard of care of NBS remains to be seen. This will be evaluated and informed by the results of research studies such as ours, and through the wider international effort and experience in this field.

## Figures and Tables

**Table 1:** Opportunities and challenges for metabolomics and genomics in newborn screening.

	Metabolomic NBS	Genomic NBS
Techniques	Targeted—LC-MS/MS ^1^ Untargeted—LC-MS/TOF ^2^ Metabolites and Lipids [8,9,10]	Targeted panel Whole-genome sequencing
Comparative screening cost	Low [11]	High [12]
Relative public acceptability	High [13]	Mixed [14,15,16]
Opportunities	DBS ^3^ for retrospective epidemiological studies [17] Building on existing NBS workflows [18,19,20] Closer to phenotype [21,22] Ability to screen multiple conditions at once [23] Possibility of new biomarker discovery [24,25]	Applicable to any condition type as a single test [26,27,28,29] Up-front molecular diagnosis [30,31,32] Lifetime re-use of data (WGS) [31,33] Enabling research into gene–disease associations, treatment developments, population variation, and pharmacogenetic variation [31,33,34,35,36]
Challenges	Feature characterization and data interpretation [37,38] May not be suitable for all condition types [23] Custom validation for each targeted condition [39,40,41] Need for sufficiently large validation cohorts [42] Results can be affected by sampling factors unrelated to conditions screened for	Consensus of which genes/variants to report [28,29,43] Possibility of identifying adult-onset conditions/variants [44,45,46] Novel variants difficult to interpret [47,48] Low pick-up for some conditions [49,50] Genetic counseling at scale [51,52] Management of large data at scale Meeting required turnaround time [53]

^1^ LC-MS/MS: liquid chromatography–tandem mass spectrometry; ^2^ LC-MS/TOF: liquid chromatography–time-of-flight mass spectrometry; ^3^ DBS: dried blood spots.

## Data Availability

No new data were created or analyzed in this study. Data sharing is not applicable to this article.
